# Efficacy of nivolumab in pre-treated non-small-cell lung cancer patients harbouring *KRAS* mutations

**DOI:** 10.1038/s41416-018-0234-3

**Published:** 2018-10-31

**Authors:** Francesco Passiglia, Federico Cappuzzo, Oscar Alabiso, Anna Cecilia Bettini, Paolo Bidoli, Rita Chiari, Carlotta Defferrari, Angelo Delmonte, Giovanna Finocchiaro, Guido Francini, Francesco Gelsomino, Diana Giannarelli, Monica Giordano, Alfonso Illiano, Lorenzo Livi, Olga Martelli, Clara Natoli, Gianfranco Puppo, Enrico Ricevuto, Elisa Roca, Daniele Turci, Domenico Galetta

**Affiliations:** 1 Department of Surgical, Oncological and Stomatological Disciplines, University of Palermo, Palermo, Italy; 2 Department of Oncology and Hematology, AUSL Romagna, Ravenna, Italy; 3 University Hospital “Maggiore della Carità”, Novara, Italy; 4 Ospedale Papa Giovanni XXIII, Bergamo, Italy; 5 Ospedale S. Gerardo, Monza, Italy; 6 Department of Medical Oncology, Santa Maria della Misericordia Hospital, University of Perugia, Perugia, Italy; 7 Ospedali Galliera, Genova, Italy; 8 Istituto Scientifico Romagnolo per lo Studio e la Cura dei Tumouri (IRST), Meldola, Italy; 9 Istituto Clinico Humanitas, Milano, Italy; 10 Policlinico Le Scotte, Siena, Italy; 11 Policlinico Sant’Orsola-Malpighi, Bologna, Italy; 12 Biostatistics Unit, Scientific Direction, IRCCS Regina Elena National Cancer Institute, Rome, Italy; 13 Ospedale “S. Anna”, Como, Italy; 14 AO del Colli, Monaldi-Cotugno-CTO, Napoli, Italy; 15 AOU Careggi, Firenze, Italy; 16 Ospedale San Giovanni Addolorata, Roma, Italy; 17 Department of Medical, Oral and Biotechnological Sciencesl, CeSI-MeT University G. D’Annunzio, Chieti-Pescara, Italy; 18 AOU Pisana, Pisa, Italy; 19 Assistenza Oncologica Territoriale, Ospedale San Salvatore, Rete Oncologica ASL1 Abruzzo, Università di L’Aquila, Aquila, Italy; 20 ASST Spedali Civili di Brescia, Brescia, Italy; 21 Ospedale “S. Maria delle Croci”, Ravenna, Italy; 22 Istituto Tumouri “Giovanni Paolo II”, Bari, Italy

**Keywords:** Non-small-cell lung cancer, Predictive markers

## Abstract

**Background:**

The present study investigated the efficacy and safety of nivolumab in pre-treated patients with advanced NSCLC harbouring *KRAS* mutations.

**Methods:**

Clinical data and *KRAS* mutational status were analysed in patients treated with nivolumab within the Italian Expanded Access Program. Objective response rate, progression-free survival and overall survival were evaluated. Patients were monitored for adverse events using the National Cancer Institute Common Terminology Criteria for Adverse Events.

**Results:**

Among 530 patients evaluated for *KRAS* mutations, 206 (39%) were positive while 324 (61%) were *KRAS* wild-type mutations. *KRAS* status did not influence nivolumab efficacy in terms of ORR (20% vs 17%, *P* = 0.39) and DCR (47% vs 41%, *P* = 0.23). The median PFS and OS were 4 vs 3 months (*P* = 0.5) and 11.2 vs 10 months (*P* = 0.8) in the *KRAS*-positive vs the *KRAS*-negative group. The 3-months PFS rate was significantly higher in the *KRAS*-positive group as compared to the *KRAS*-negative group (53% vs 42%, *P* = 0.01). The percentage of any grade and grade 3–4 AEs were 45% vs 33% (*P* = 0.003) and 11% vs 6% (*P* = 0.03) in *KRAS*-positive and *KRAS-*negative groups, respectively.

**Conclusions:**

Nivolumab is an effective and safe treatment option for patients with previously treated, advanced non-squamous NSCLC regardless of *KRAS* mutations.

## Background

The advent of immune-checkpoint inhibitors (ICIs) in clinical practice^1^ is leading to a significant improvement of life expectancy in patients with advanced non-small-cell lung cancer (NSCLC). Modulating the antitumour immune response by targeting the programmed cell death 1 (PD-1)/programmed cell death 1 ligand (PD-L1) axis emerged as an effective and tolerable treatment in early phase I studies,^2^ offering the potential for durable disease control and long-term survival outcomes in heavily pre-treated NSCLC patients.^3^ Four randomised phase III trials have subsequently demonstrated that single-agent ICIs, nivolumab,^4,5^ pembrolizumab^6^ or atezolizumab^7^ significantly improved overall survival (OS) and quality of life (QoL) as compared to docetaxel in pre-treated NSCLC patients, emerging as new standard of care in second or later lines of therapy. In particular, the phase III Checkmate 057 trial^5^ first demonstrated a significant superiority in terms of objective response rate (ORR), OS, tolerability and QoL in favour of the anti-PD-1 nivolumab over docetaxel in pre-treated patients with non-squamous NSCLC. Importantly, nivolumab resulted superior to docetaxel irrespective of tumour PD-L1 expression, even if higher efficacy was detected among patients expressing high PD-L1 levels. Landmark survival analysis^8^ excluding patients with poor prognosis who died within the first 3 months of therapy, has subsequently demonstrated that patients with low or no tumour PD-L1 expression who were alive at 3 months also benefited from nivolumab. These evidences led to the final approval of nivolumab by regulatory authorities in March 2016 for the second/third-line treatment of non-squamous NSCLC regardless of tumour PD-L1 expression.^9^ The data emerging from both randomised trials^6,7,10^ and real-life experiences^11,12^ suggested that immunotherapy is effective in a significant subgroup of patients, leading to durable disease control, long-term survival and improved QoL. Conversely, about 50% of pre-treated patients did not gain any benefit from ICIs and a small subgroup of them developed early progression or death within 3 months of therapy.^8,11–15^ Thus, identifying predictive biomarkers of clinical response/resistance to ICIs is crucial for the selection of an appropriate candidate to immunotherapy. Looking for molecular predictors of ICI efficacy, the pre-specified subgroup analysis of the 057 trial^10^ clearly showed that nivolumab did not improve neither PFS nor OS in patients with *epidermal growth factor receptor (EGFR)*-positive NSCLC. Similar results were obtained in trials with pembrolizumab or atezolizumab as well as in recent meta-analysis.^16,17^

*KRAS* mutations represent the most common oncogene driver detected in about 30% of non-squamous NSCLC, usually occurring at codons 12–13 and associated with cigarette smoking.^18,19^ Several efforts have been made by the scientific community to understand the potential role of *KRAS* mutations as a therapeutic target in cancer cells, but no effective KRAS-inhibitors have been approved yet for clinical use. The efficacy of nivolumab in *KRAS*-mutated NSCLC is not well defined. The results from clinical trials suggested that patients harbouring *KRAS*-mutations could result in more sensitivity to nivolumab as compared to *KRAS* wild-type mutations, but the small number of patients evaluated in single trials^7,10^ precluded any definitive conclusion. A recent meta-analysis^17^ investigated the predictive role of *KRAS*-mutations in 519 patients with previously treated NSCLC included in trials with nivolumab or atezolizumab (CheckMate 057, OAK and POPLAR studies). The results of such analysis showed a greater benefit in *KRAS*-mutant subgroups even if the difference was not statistically significant, likely because *KRAS*-mutation status was known only in a small fraction of cases. In the present study, we investigated whether nivolumab could result effective in terms of ORR, PFS and OS in pre-treated metastatic NSCLC patients harbouring *KRAS* mutations.

## Methods

### Patients

The study was conducted in patients participating in the Italian expanded access program (EAP). Patients were eligible if they aged ≥18 years, had histologically or cytologically confirmed diagnosis of non-squamous NSCLC, stage IIIB–C/IV (according to Version 8 of the International Association for the Study of Lung Cancer (IASLC) TNM Staging System), Eastern Cooperative Oncology Group (ECOG) performance-status score <3, and had disease progression or recurrence after receiving at least one prior systemic therapy for advanced/metastatic disease.

Patients were excluded if they had autoimmune disease, symptomatic interstitial lung disease, systemic immunosuppression and prior treatment with immune-stimulatory antitumour agents including checkpoint-inhibitors. Patients with brain metastases were eligible if they have received prior loco-regional treatment and were stable at the time of inclusion. Tumour PD-L1 status was not required.

The study was conducted in accordance with the International Conference on Harmonization Guidelines on Good Clinical Practice and the Declaration of Helsinki. The trial protocol was previously approved by the Independent Ethics Committee and all the patients provided a written informed consent before enrolment.

### Study design and treatment

We retrospectively collected clinical data and *KRAS* mutational status from patients’ charts and hospital electronic medical records for eligible patients who have been treated with nivolumab at 168 Italian cancer centres from May 2015 to December 2016. All included patients were followed until the end of data collection on September 2017.

Nivolumab was available upon physicians’ request for eligible patients through the EAP. Nivolumab 3 mg/kg was administered intravenously every 2 weeks for ≤24 months. Patients included in the analysis received ≥1 dose of nivolumab. The treatment was continued until disease progression or unacceptable toxicity, or the completion of permitted cycles (≤24 months).

*KRAS* mutation testing data from tumour samples obtained before enrolment in the EAP were used where available. DNA extracted from tissue/cytological samples was subjected to *KRAS* mutational analysis using local practices.

Radiological evaluation of treatment efficacy by CT-scan was performed at week 12 and every 12 weeks thereafter until disease progression and responses were evaluated by Response Evaluation Criteria in Solid Tumours (RECIST) v1.1. Patients were monitored for AEs using the National Cancer Institute Common Terminology Criteria for Adverse Events v4.0.

### Statistical analysis

The main objective of the study was to assess nivolumab efficacy in terms of ORR, PFS and OS in NSCLC patients with or without *KRAS* mutations. Secondary endpoint was to assess whether nivolumab safety profile was different in individuals with or without *KRAS* mutations.

For efficacy analysis, patients were grouped according to their tumour *KRAS* mutational status into ‘positive’ if they harboured *KRAS* mutations or ‘negative’ if they did not have *KRAS* mutations. Patients’ clinical–pathological characteristics and associations with *KRAS* mutational status were examined with a descriptive analysis comparing the differences by *χ*^2^ test or Fisher’s exact test as appropriate.

Investigator-assessed efficacy outcomes, including ORR, disease control rate (DCR), median PFS and OS, were assessed in *KRAS*-positive vs *KRAS*-negative patients both in the overall population and in predefined subgroups of patients. ORR was defined as the combined rates of complete response (CR) and partial response (PR). DCR was defined as the combined rates of CR, PR and stable disease (SD). Median PFS was defined as the time between the date of inclusion and the date of disease progression determined by RECIST v1.1, death from any cause or the last follow-up. Median OS was defined as the time between the date of inclusion and the date of death. Survival analysis was performed using Kaplan–Meier method, providing median and *P*-values, with the use of the log-rank test for comparisons. Univariate and multivariate analyses were performed using the logistic regression model when referring to binary outcome and Cox regression model when considering time to events. A *P*-value < 0.05 was used as a threshold for statistical significance.

All statistical analyses were performed with SPSS Statistics software version 21 (IBM, Armonk, New York, USA).

## Results

### Patients’ characteristics

From May 2015 to December 2016, a total of 1588 non-squamous NSCLC patients were considered eligible and participated in the EAP at 168 centres in Italy. Among 530 patients evaluated for *KRAS* mutations, 206 (39%) resulted positive while 324 (61%) were *KRAS* wild-type mutations. *KRAS* mutation subtypes were not known in the analysed population. Epidermal growth factor receptor (EGFR)-activating mutations were detected in 17/324 patients (5%), while ALK/ROS1 rearrangements were found in 7/324 (2%) of *KRAS* wild-type patients. As reported in Table 1, the baseline characteristics were similar between the two subgroups. However, in the *KRAS*-positive group, the percentage of current/former smokers was significantly higher than in the *KRAS*-negative group (86% vs 76%, *P* = 0.01), as was the percentage of patients with CNS metastasis (29% vs 20%, *P* = 0.01). Patients with *KRAS* mutations received a median of eight doses (range: 1–54), with a median follow-up of 8.0 months (range: 0.1–25.9), while *KRAS*-negative patients received a median of seven doses (range: 1–52), with a median follow-up of 7.4 months (range: 0.2–27.4).

**Table 1:** Baseline patients’ characteristics

Characteristic	KRAS-positive (*n* = 206)	KRAS-negative (*n* = 324)	*P*-value
Median age, years (range)	66 (36–87)	65 (29–86)	0.09
Male, *n* (%)	129 (63)	218 (67)	0.27
Smoking status, *n* (%)			**0.01**
Smoker	45 (24)	78 (25)	
Former smoker	119 (62)	157 (51)	
Never-smoker	27 (14)	75 (24)	
Unknown	15	14	
ECOG PS, *n* (%)			
0	80 (39)	132 (41)	0.88
1	111 (54)	167 (52)	
2	14 (7)	21 (7)	
Unknown	1	4	
Metastatic site, *n* (%)			
CNS	60 (29)	64 (20)	**0.01**
Liver	35 (17)	77 (24)	0.06
Number of prior therapies, *n* (%)			
1	89 (43)	125 (39)	0.49
2	52 (25)	97 (30)	
3	41 (20)	57 (18)	
≥4	24 (14)	44 (14)	

*n* number of patients, *PS* performance status, *CNS* central nervous system. Bold values indicate statistical significance

### KRAS mutations and tumour response

Among patients with *KRAS*-positive NSCLC treated with nivolumab, one patient (0.5%) experienced CR, 39 (19%) had a partial response (PR), 55 (27%) stable disease (SD) and 88 (43%) progressive disease (PD). No significant differences between *KRAS*-positive and *KRAS*-negative patients have been observed as regards both ORR (20% vs 17%, *P* = 0.39) and DCR (47% vs 41%, *P* = 0.23), as illustrated in Table 2. The results of both ORR and DCR analyses across different predefined subgroups of patients were consistent with those observed in the overall population.

**Table 2:** Response to treatment

Response, *n* (%)	KRAS-positive (*n* = 206)	KRAS-negative (*n* = 324)	*P*-value
ORR	41 (20)	55 (17)	0.39
DCR	96 (47)	134 (41)	0.23
Best overall response			
CR	2 (1)	1 (<1)	
PR	39 (19)	54 (17)	
SD	55 (27)	79 (24)	
PD	88 (43)	153 (47)	
Death	12 (5)	30 (9)	
Not determined	10 (5)	7 (2)	

*n* number of patients, *ORR* objective response rate, *DCR* disease control rate, *CR* complete response, *PR* partial response, *SD* stable disease, *PD* progression disease

### KRAS mutations and patients’ survival

**Fig. 1:**
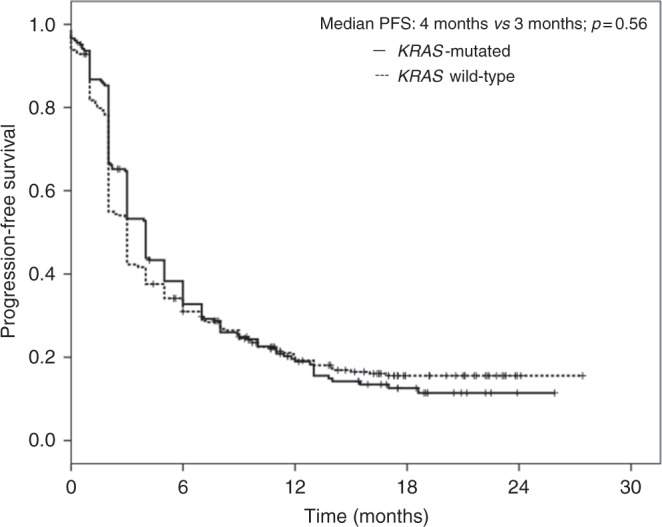
Kaplan–Meier analysis of progression-free survival (PFS) in NSCLC patients according to the *KRAS-*mutation status

As illustrated in Fig. 1, at the time of survival analysis (median follow-up of 8.1 months, range: 0.1–27.4), median PFS was 4 months (95% CI: 3.6, 4.4) in the *KRAS*-positive group and 3 months (95% CI: 3.6–4.4) in the *KRAS*-negative group (*P* = 0.56). The 3-months PFS rate was significantly higher in the *KRAS*-positive group as compared to the *KRAS*-negative group (53% vs 42%, *P* = 0.01). The 6-months and 12-months PFS rate was similar between the two groups. The 6-months rate of PFS was 33% in the *KRAS*-positive group and 31% in the *KRAS-*negative group (*P* = 0.63); the 12-months rate of PFS was 19% in both the groups (*P* = 0.99).

**Fig. 2:**
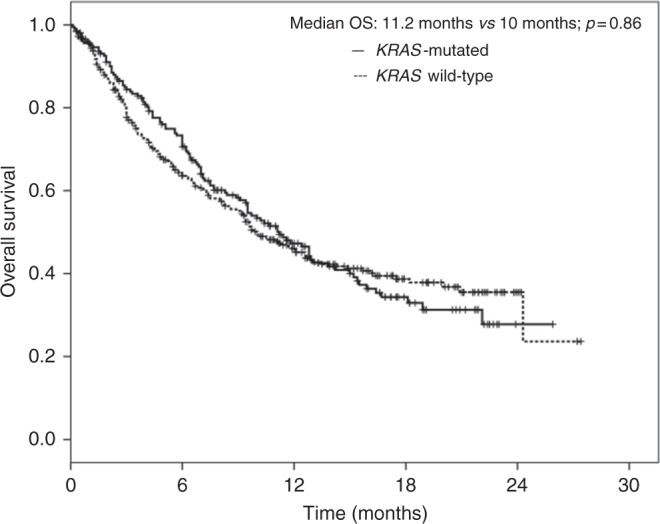
Kaplan–Meier analysis of overall survival (OS) in NSCLC patients according to the *KRAS-*mutation status

There were no significant OS differences between *KRAS*-positive and *KRAS*-negative patients. The median OS was 11.2 months (95% CI: 9.3–13.1) in the *KRAS*-positive group and 10 months (95% CI: 9.3–13.1) in the *KRAS*-negative group (*P* = 0.86, Fig. 2). The 3-months rate of OS was 84% in the *KRAS*-positive group and 78% in the *KRAS*-negative group (*P* = 0.09); the 6-months rate of OS was 71% in the *KRAS*-positive group and 64% in the *KRAS*-negative group (*P* = 0.09); the 12-months rate of OS was 47% in the *KRAS*-positive group and 46% in the *KRAS*-negative group (*P* = 0.92). The results of ORR, PFS and OS analyses across different predefined subgroups of patients were consistent with those observed in the overall population.

### Safety

Treatment-related AEs (TRAEs) of any grade and grade 3–4 for *KRAS*-positive and *KRAS*-negative non-squamous NSCLC patients treated with nivolumab are shown in Table 3. The percentage of patients with TRAEs of any grade was significantly higher in the *KRAS*-positive group (45% vs 33%, *P* = 0.003) as was the percentage of patients with TRAEs of grade 3–4 (11% vs 6%, *P* = 0.03). The percentage of patients who discontinued treatment was 81% in *KRAS*-positive and 84% in *KRAS*-negative patients. TRAEs leading to discontinuation were hepatic, gastrointestinal and pulmonary with similar frequencies observed in the different subgroups of patients. No treatment-related deaths have been reported. The results of safety analyses across different predefined subgroups of patients were consistent with those observed in the overall population.

**Table 3:** Treatment-related adverse events (TRAEs)

Event	KRAS-positive (*n* = 206)		KRAS-negative (*n* = 324)	
	Any grade, *n* (%)	Grade 3–4, *n* (%)	Any grade, *n* (%)	Grade 3–4, *n* (%)
Any treatment-related AE	93 (45)	22 (11)	108 (33)	18 (6)
General				
Fatigue/asthenia	39 (19)	9 (4)	34 (10)	8 (2)
Pyrexia	12 (6)	0	11 (3)	0
Anorexia	11 (5)	1 (<1)	18 (6)	0
Skin and mucosal	16 (8)	1 (<1)	22 (7)	3 (1)
Rash	2 (1)	0	9 (3)	1 (<1)
Gastrointestinal	35 (17)	4 (2)	41 (13)	4 (2)
Diarrhoea	8 (4)	1 (<1)	16 (5)	1 (<1)
Nausea/vomiting	11 (5)	1 (<1)	12 (4)	1 (<1)
Haematologic	12 (6)	2 (1)	6 (2)	3 (1)
Anaemia	6 (3)	2 (1)	5 (2)	3 (1)
Pain	19 (9)	2 (<1)	19 (6)	1 (<1)
Hepatic/pancreatic	14 (7)	43 (2)	15 (5)	2 (1)
Increased transaminase	7 (3)	2 (1)	3 (1)	0
Increased lipase/amylase	1 (<1)	0	2 (1)	0
Endocrine	10 (5)	1 (<1)	18 (6)	1 (<1)
Hypothyroidism	5 (2)	1 (<1)	9 (3)	0
Hyperthyroidism	5 (2)	0	7 (2)	0
Autoimmune hypophysitis	0	0	0	0
Respiratory/pulmonary	36 (17)	4 (2)	43 (13)	6 (2)
Dyspnoea	13 (6)	3 (1)	18 (6)	2 (1)
Pneumonitis	6 (3)	1 (<1)	8 (2)	3 (1)

*n* number of patients, *AE* adverse event, *TRAEs* treatment-related adverse events

### Multivariable analysis

Multivariable Cox proportional regression analysis was performed to assess whether *KRAS* mutations were independent factors related to nivolumab safety in terms of any grade and grade 3–4 toxicities. All clinical–pathological parameters found to have a *P*-value < 0.05 at univariate analysis were included as covariates in the multivariable model.

KRAS mutations remained significantly associated with higher toxicity rate, including both any grade AEs (OR: 1.66 (1.14–2.41) *P* = 0.008) and grade 3–4 AEs (OR: 2.25 (1.14–4.44) *P* = 0.02) (Table 4).

**Table 4:** Univariable and multivariable analysis

Parameter	Any grade TrAEs		G3–4 TrAEs	
	Univariable OR (95% CI); *P*	Multivariable OR (95% CI); *P*	Univariable OR (95% CI); *P*	Multivariable OR (95% CI); *P*
KRAS status (mut vs wt)	0.57 (0.35–0.91) *P* = 0.02	1.66 (1.14–2.41) *P* = 0.008	0.74 (0.30–1.83) *P* = 0.52	2.03 (1.06–3.89) *P* = 0.03
Age (years)	0.99 (0.97–1.01) *P* = 0.17	—	0.97 (0.94–1.01) *P* = 0.10	—
Sex	0.82 (0.57–1.18) *P* = 0.29	—	0.98 (0.50–1.92) *P* = 0.95	—
ECOG PS (2 vs 0–1)	0.74 (0.35–1.54) *P* = 0.41	—	0.73 (0.17–3.16) *P* = 0.67	—
Smoking habits (never vs current/former)	1.65 (1.15–2.36) *P* = 0.006	0.59 (0.36–0.95) *P* = 0.03	2.03 (1.06–3.89) *P* = 0.03	—
Brain metastasis (yes vs no)	1.24 (0.82–1.87) *P* = 0.30	—	1.44 (0.71–2.93) *P* = 0.31	—
Liver metastasis (yes vs no)	0.62 (0.40–0.98) *P* = 0.04	—	0.78 (0.33–1.81) *P* = 0.56	—
Previous lines (>1 vs 1)	0.82 (0.57–1.17) *P* = 0.27	—	0.82 (0.43–1.56) *P* = 0.54	—

*n* number of patients, *TRAEs* treatment-related adverse events, *OR* odds ratio, *95% CI* confidence intervals, *P* *P*-value, *PS* performance status, *mut* mutated, *wt* wild-type

## Discussion

To the best of our knowledge, this is the largest study investigating the predictive role of *KRAS* mutations in advanced non-squamous NSCLC treated with nivolumab. The results of this real-world analysis demonstrated that both clinical efficacy and safety of nivolumab were comparable to those observed in the phase III randomised CheckMate 057 trial, including the same NSCLC population.^5^

Our results demonstrated that *KRAS* status is not a reliable predictor of nivolumab efficacy in terms of RR, PFS and OS. Differences in all clinical endpoints were not statistically significant, with the only exception of 3-months PFS that was significantly higher in the *KRAS*-positive group.

Interest in *KRAS* mutant NSCLC is growing because of the lack of any specific agent available in patients harbouring such molecular alteration, the high incidence in non-squamous NSCLC and the association with smoking history and therefore with tumour mutational burden, one of the most innovative predictive biomarkers to immunotherapy. Preclinical and clinical evidences suggested that *KRAS*-positive NSCLC seems to gain more benefit from immunotherapy. First of all, *KRAS*-mutant tumours are characterised by the presence of CD8 + lymphocyte infiltrates in the tumour microenvironment (TME),^20^ while a significant association between *KRAS* mutations and PD-L1 expression has been observed in lung adenocarcinoma.^21,22^ Coelho et al.^23^ have recently demonstrated that oncogenic RAS signalling upregulated tumour PD-L1 expression stabilising the PD-L1 transcript in *KRAS*-mutant adenocarcinoma, thus providing an additional mechanism whereby *KRAS*-positive tumours respond to PD-1/PD-L1 inhibitors. Recent studies demonstrated how the crosstalk between the cancer cells intrinsic RAS signalling and the TME extended beyond the tumour PD-L1 expression, regulating many other different TME components,^24^ such as inflammatory cells, immune T-cells and myeloid cells density, cancer-associated fibroblasts and endothelial cells properties and extracellular matrix (ECM) composition, ultimately favouring immune-escape, cancer growth and metastatic process. The possibility of using *KRAS* status for selecting patients potentially sensitive to immunotherapy is certainly of great interest. Indeed, this biomarker is generally included among the molecular tests performed in metastatic NSCLC, facilities are available in the majority of centres, it is relatively easy to perform and not expansive. However, it is now clear that *KRAS*-mutant NSCLC is a heterogeneous disease, including different tumour subtypes with variable biological background, different prognosis and clinical response to immunotherapy. A recent work^25^ showed that tumours with co-occurring *KRAS/P53* mutations were associated with higher PD-L1 expression as well as elevated PD-L1+/CD8+ cell ratio and increased mutation burden as compared to tumours with *KRAS* or *P53* single mutation. Interestingly, patients with *KRAS*+*/P53*+ NSCLC also showed a remarkable and durable clinical benefit from anti-PD-1 therapy, suggesting a synergistic and complementary effect of both signalling pathways to the TME immunogenicity. Conversely co-occurring inactivation of *LKB1/STK11* tumour suppressor gene was associated with lack of tumour response and shorter PFS and OS as compared to *LKB1/STK11* wild-type patients with *KRAS*-mutant lung adenocarcinoma, suggesting *LKB1*-loss as a major driver of immune-escape and a genomic biomarker of innate resistance to ICIs.^26^ Previous reports described as the inactivation of *LKB1* were associated with lower PD-L1 expression levels and paucity of infiltrating CD8+ lymphocytes in the TME of *KRAS*-positive NSCLC,^27^ suggesting that normal *LKB1* tumour suppressor gene plays a crucial role in maintaining a *KRAS*-mutant-driven immunosuppressive TME. Recent findings demonstrated that STK11/LKB1 alterations are associated with lack of response to PD-1 inhibitors efficacy, regardless of *KRAS* mutations or PD-L1 expression status,^26,28^ suggesting that different combinations of P53, STK11 and EGFR mutations were associated with different tumour microenvironments and may predict clinical response to PD-1 blockade.^28^ Unfortunately, neither *P53* nor *LKB1* status were known for *KRAS*-positive NSCLC patients included in our study because of the lack of tumour tissue available for molecular analysis. Since only 24/324 *KRAS* wild-type patients had EGFR-activating mutations or ALK/ROS1 rearrangements, we were not able to evaluate their impact on efficacy outcomes observed with nivolumab in the overall population. Further trials including larger cohorts of NSCLC patients with known *KRAS, P53, STK11, EGFR* and PD-L1 status are warranted.

Interestingly, our series provided new information with regard to nivolumab tolerability according to tumour *KRAS*-mutation status in a real-life setting. Although nivolumab was overall well tolerated, the percentage of both any grade and grade 3–4 TRAEs was significantly higher in the *KRAS*-positive group, suggesting a potential interaction between KRAS-signalling and treatment tolerability. The reasons for this observation remain speculative and warrant further investigation in clinical studies. However, imbalances in the clinical characteristics of patients at baseline may have influenced the different tolerability profile of nivolumab between the two treatment groups.

In conclusion, the results of this study showed that KRAS mutations are not useful for selecting patients candidate to nivolumab therapy. Nivolumab is an effective and safe treatment option for patients with previously treated, advanced NSCLC regardless of *KRAS*-mutation status.

## Availability of data and material

All the data analysed supporting the results reported in the article may be found/ are archived at the Biostatistics Unit, Scientific Direction, IRCCS Regina Elena National Cancer Institute, Rome.
